# Longitudinal Imaging Studies of Tumor Microenvironment in Mice Treated with the mTOR Inhibitor Rapamycin

**DOI:** 10.1371/journal.pone.0049456

**Published:** 2012-11-20

**Authors:** Keita Saito, Shingo Matsumoto, Hironobu Yasui, Nallathamby Devasahayam, Sankaran Subramanian, Jeeva P. Munasinghe, Vyomesh Patel, J. Silvio Gutkind, James B. Mitchell, Murali C. Krishna

**Affiliations:** 1 Radiation Biology Branch, Center for Cancer Research, National Cancer Institute, National Institutes of Health, Bethesda, Maryland, United States of America; 2 Laboratory of Radiation Biology, Department of Environmental Veterinary Sciences, Graduate School of Veterinary Medicine, Hokkaido University, Sapporo, Japan; 3 National Institute of Neurological Disorder and Stroke, National Institutes of Health, Bethesda, Maryland, United States of America; 4 National Institute of Dental and Craniofacial Research, National Institutes of Health, Bethesda, Maryland, United States of America; The University of Hong Kong, Hong Kong

## Abstract

Rapamycin is an allosteric inhibitor of mammalian target of rapamycin, and inhibits tumor growth and angiogenesis. Recent studies suggested a possibility that rapamycin renormalizes aberrant tumor vasculature and improves tumor oxygenation. The longitudinal effects of rapamycin on angiogenesis and tumor oxygenation were evaluated in murine squamous cell carcinoma (SCCVII) by electron paramagnetic resonance imaging (EPRI) and magnetic resonance imaging (MRI) to identify an optimal time after rapamycin treatment for enhanced tumor radioresponse. Rapamycin treatment was initiated on SCCVII solid tumors 8 days after implantation (500–750 mm^3^) and measurements of tumor pO_2_ and blood volume were conducted from day 8 to 14 by EPRI/MRI. Microvessel density was evaluated over the same time period by immunohistochemical analysis. Tumor blood volume as measured by MRI significantly decreased 2 days after rapamycin treatment. Tumor pO_2_ levels modestly but significantly increased 2 days after rapamycin treatment; whereas, it decreased in non-treated control tumors. Furthermore, the fraction of hypoxic area (pixels with pO_2_<10 mm Hg) in the tumor region decreased 2 days after rapamycin treatments. Immunohistochemical analysis of tumor microvessel density and pericyte coverage revealed that microvessel density decreased 2 days after rapamycin treatment, but pericyte coverage did not change, similar to what was seen with anti-angiogenic agents such as sunitinib which cause vascular renormalization. Collectively, EPRI/MRI co-imaging can provide non-invasive evidence of rapamycin-induced vascular renormalization and resultant transient increase in tumor oxygenation. Improved oxygenation by rapamycin treatment provides a temporal window for anti-cancer therapies to realize enhanced response to radiotherapy.

## Introduction

Multiple genetic and epigenetic events are known to result in the dysregulation of several signaling pathways that have an impact on neoplastic disease progression, such as squamous cell carcinomas (SCC) [1], [2]. One such pathway, the phosphatidylinositol 3-kinase (PI3K)-Akt pathway is frequently activated in many cancers, and controls cellular metabolism, growth, and proliferation [3]–[6]. The mammalian target of rapamycin (mTOR) is an atypical serine/threonine kinase, which acts downstream of PI3K/Akt and, therefore has become an attractive therapeutic target [7]–[10]. It follows that inhibitors of mTOR, such as rapamycin and its derivatives are currently being evaluated for molecular targeted therapy of neoplastic diseases [9].

The inhibition of mTOR with its specific allosteric inhibitor, rapamycin, provokes a rapid death of squamous xenografts, resulting in tumor regression [11]. The molecular basis of this is currently an active area of research [12]. For example, a recent study using a reverse-pharmacology approach, which involved the expression of a rapamycin-insensitive form of mTOR in squamous cancer cells, showed that cancer cells are the primary targets of rapamycin *in vivo*, and that mTOR controls the expression of hypoxia-inducible factor-1α (HIF-1α), a key transcription factor that orchestrates the cellular response to hypoxic stress, including the regulation of the expression of angiogenic factors, thus providing a likely mechanism by which rapamycin exerts its tumor suppressive and antiangiogenic effects [13]. Blocking mTOR pathway in SCC tumors was also shown to prevent accumulation of HIF-1α resulting in inhibition of processes involved in glucose metabolism as well as decrease in pro-angiogenic factors such as vascular endothelial growth factor (VEGF) [13].

Recent studies using magnetic resonance imaging (MRI) showed that treatment with mTOR inhibitors results in strong antiangiogenic and anti-vascular effects in solid tumors [12]. Although there are distinctions between the effects of mTOR inhibitors and antiangiogenic agents on tumor vasculature, it was suggested that rapamycin induced antiangiogenic effects also mediate vascular re-normalization as in the case of conventional antiangiogenic agents [14]. Since vascular normalization improves tumor oxygenation as well as delivery of therapeutic drugs [15]–[19], examining whether such a process occurs in the case of mTOR inhibitors may explain the efficacy of rapamycin's radiosensitizing effects [20]. If such a temporal change of tumor oxygenation can be identified for rapamycin by using a non-invasive pO_2_ mapping technique such as by electron paramagnetic resonance imaging (EPRI) it becomes then possible to appropriately schedule the two modalities for better therapeutic outcomes.

Electron paramagnetic resonance (EPR) is a spectroscopic technique similar to nuclear magnetic resonance. EPR detects paramagnetic species that have unpaired electrons such as transition metal complexes and free radicals. With the recent availability of triarylmethyl radical probes (TAM) as in vivo compatible paramagnetic tracers, EPRI is now being explored for mapping tissue oxygen in live animals [21]–[24]. The fundamental basis for EPRI in monitoring tissue oxygen using TAM stems from the paramagnetic nature of molecular oxygen arising from its two unpaired electrons. The collisional interaction between TAM and dissolved paramagnetic oxygen leads to a broadening of the spectral line width of TAM. The EPR spectral broadening of TAM is linear with oxygen concentration, providing quantitative capability of EPR in determining tissue pO_2_ [22], [23]. Furthermore, utilizing magnetic field gradients as in MRI, the spatial distribution of the TAM tracer can be obtained in a living subject. By extracting the pO_2_ dependent EPR line widths, a three-dimensional pO_2_ map can be generated with a spatial resolution of 1.5–2 mm^3^ in only 3–10 min [23]. The technique can be used to longitudinally monitor changes in pO_2_ on the same animal [19], [21], [24]. While images from EPRI provide maps of pO_2_, they lack the anatomic detail as provided by MRI scans. We therefore designed a combined EPRI+MRI system operating at a common frequency of 300 MHz in both modalities with the corresponding magnetic fields at 10 mT (EPRI) and 7 T (MRI). Sequential scans with the two modalities employing a common resonator enable obtaining pO_2_ maps with anatomic guidance. Additional information gathered from MRI such as blood volume, enable to achieve a more complete understanding of tumor physiology.

In this report, pO_2_ and microvessel density in SCC tumors were longitudinally monitored by using EPRI and MRI to elucidate rapamycin effect on tumor oxygenation and angiogenesis in vivo.

## Methods

### Ethics Statement

All animal experiments were carried out in compliance with the *Guide for the care and use of laboratory animal resources* (National Research Council, 1996) and approved by the National Cancer Institute Animal Care and Use Committee (NCI-CCR-ACUC (Bethesda), Protocol# RBB-155 and 159).

### Cell Culture and Western Blot Analysis

SCCVII cell line was kindly obtained from Dr. T. Philips, University of California San Francisco (San Francisco, CA), and was tested in 2011 by IDEXX RADIL (Columbia, MO) using a panel of microsatellite markers. The SCCVII is a squamous carcinoma which arose spontaneously in the abdominal wall of a C3H mouse in the laboratory of Dr. H. Suit, Massachusetts General Hospital (Boston, MA) [25], [26], and was subsequently adapted for clonogenic growth by Dr. K. Fu, University of California San Francisco [27].

SCCVII cells were initially grown in RPMI supplemented with 10% FCS to 70% confluency, and following overnight serum starvation, cells were treated with 100 nM concentration of rapamycin (LC Laboratories) for the indicated time. Exposure to Epidermal growth factor (EGF; Sigma Aldrich) was used as a positive control at 100 ng/mL for 30 min. After treatment, cells were lysed and total cellular proteins were processed for western blot analysis for the indicated proteins and appropriate antibodies (Cell Signaling; GAPDH was from Santa Cruz).

### Animals

Female C3H/Hen mice were supplied by the Frederick Cancer Research Center, Animal Production (Frederick, MD). SCCVII solid tumors were formed by injecting 5×10^5^ SCC cells subcutaneously into the right hind leg of C3H mice. The experiment was initiated 8 days after tumor cells implantation. The tumor size during experiments was 550–1500 mm^3^ (the tumor volume (V = length×width^2^×π/6)). Body weight measured before the experiments was 21–27 g. Mice were anesthetized by isoflurane (4% for induction and 1.5% for maintaining anesthesia) in medical air (750 mL/min) and positioned prone with their tumor-bearing legs placed inside the resonator. During EPRI and MRI measurements, the breathing rate of the mouse was monitored with a pressure transducer (SA Instruments Inc.) and maintained at 60±10 breaths per minute. Core body temperature was also monitored with a non-magnetic rectal temperature probe (FISO) and maintained at 37±1°C with a flow of warm air. For administration of TAM and ultrasmall superparamagnetic iron oxide (USPIO, Molday ION from BioPal Inc., Worcester, MA) solutions, a 30-gauge needle was cannulated into the tail vein and extended using polyethylene tubing (PE-10).

### Rapamycin treatment

Rapamycin (LC laboratories, Woburn, MA) was dissolved in ethanol, and further diluted in an aqueous solution of 5.2% Tween 80 and 5.2% polyethylene glycol immediately before use. Rapamycin was injected intraperitoneally to tumor bearing mice at a dose of 5 or 10 mg/kg body weight/day, and an equal volume of diluent was injected to control groups. The treatment was started 8 days after tumor implantation, and the schedule was a single injection per mouse, per day, consecutively during experiments.

### EPR imaging

Technical details of the EPR scanner operating at 300 MHz, data acquisition based on the single-point imaging (SPI) modality, image reconstruction, and the oxygen mapping procedure were described in earlier reports [22], [23], [28]–[30]. After the animal was placed in the resonator, TAM (Ox063, GE Healthcare) was injected intravenously as a 1.125 mmol/kg bolus through the cannula placed in the tail vein. EPR signals were collected following the RF excitation pulses (60 ns, 80 W, 70° flip angle) using an analog digital converter (200 megasamples/s). The repetition time (TR) was 6.0 µs. The FIDs were collected under a nested looping of the x, y, z gradients and each time point in the FID underwent phase modulation enabling 3D spatial encoding. Since FIDs last for a couple of microseconds, it is possible to generate a sequence of T_2_^*^ mapping, which allowed pixel-wise estimation of in vivo pO_2_. The spatial resolution of pO_2_ images measured using EPRI was 1.8 mm, although the pixel resolution was digitally enhanced in order to co-register with MRI images.

### MRI and co-registration of pO_2_ images with anatomic images

A parallel coil resonator (17 mm i.d. and 25 mm long) with Q switch was constructed for sequential EPR and MR imaging of the tumor bearing leg. The basic description of the parallel coil resonator used for pulsed EPR and 7 T MRI operating at 300 MHz was described in an earlier report [30]. Since required quality factor (Q value) is different between EPRI and MRI, switching of Q values of the coil was done by isolating the damping resistance from the main circuit [28].

MRI scans were conducted using a 7 T scanner controlled with ParaVision 5.0 (Bruker BioSpin MRI GmbH). After a quick assessment of the sample position by a fast low-angle shot (FLASH) tripilot sequence, T_2_^*^-weighted anatomical images were obtained using a fast spin echo sequence (RARE) with an echo time (TE) of 13 ms, TR of 2,500 ms, 14 slices, RARE factor 8, resolution of 0.11×0.11 mm, and acquisition time of 80 s. For convenience of coregistration with EPRI, all MRI images had the same FOV of 2.8 cm and slice thickness of 2 mm. Blood volume calculation was performed as described previously [31]. Briefly, this technique was based on the T2* shortening effect and the consequent signal loss by USPIO injection. Spoiled gradient echo (SPGR) sequence images were collected as follows: matrix, 256×256; TE, 5.0 ms; TR, 261.5 ms; slice thickness, 2 mm; scan time, 2 min 14 sec. These images were obtained before and 5 min after USPIO injection (1.2 µL/g body weight). Percentage of tumor blood volume was estimated by the expression 100×(S_pre_−S_post_)/[S_pre_+S_post_ (W_b_/W_t_−1)], where S_pre_ and S_post_ were the signal intensities of each voxel before and after USPIO injection and W_b_ and W_t_ were the intra- and extravascular water fractions. Dynamic contrast enhanced (DCE)-MRI study was carried out using a 1 T scanner (Bruker ICON). For T1 mapping, coronal RARE images of three slices passing through the tumor region were obtained with TR values of 500, 1000, and 3000 ms. Gd-DTPA solution (50 mM, 5 µL/g body weight) was intravenously injected into tail vein of mouse 2 min after start of the fast gradient echo scans. The scan parameters are as follows: TE = 6 ms, TR = 118 ms, tip angle 30°, 2 mm thickness×4 slices, 15 sec acquisition time per image, and 60 repetition. Co-registration of EPR and MRI images was accomplished using code written in MATLAB (Mathworks) as described in a previous report [23], [32].

### Immunohistochemical analysis

Tumor-bearing mice were euthanized, and tumor tissues were removed from mice. Tumor tissues were fixed with 4% paraformaldehyde and frozen using ultracold ethanol. Frozen tumors were sectioned to 10 mm thick using a cryostat, and the sections were thaw-mounted on glass slides. After blocking non-specific binding sites with Protein Block Serum-Free reagent (Dako North America Inc., Carpinteria, CA), the slides were covered by CD31 antibody (BD Biosciences, San Jose, CA; 1∶250) combined with αSMA antibody (Abcam Inc., Cambridge, MA; 1∶250) overnight at 4°C. The sections were incubated with Alexa Fluor 488 anti-rat and Alexa Fluor 555 anti-rabbit secondary antibody (Invitrogen, Carlsbad, CA; 1∶500). Then they were mounted on Prolong Gold antifade reagent with DAPI (Invitrogen). Fluorescence microscopic observation was performed using an Axiovert 200 inverted fluorescent microscope (Carl Zeiss).

The quantification of CD31 and αSMA was performed according to the method described by Zhou et al. [18]. Briefly, tissue sections were viewed at 200× magnification and more than three fields per section were captured using Image-Pro Plus Ver. 4.0 imaging software. Then the quantification of vascular density and pericyte density on each image was performed with histogram analysis using the ImageJ software package (http://rsb.info.nih.gov/ij/) and shown as the total number of positive pixels per field.

Paraformaldehyde fixed tissues were paraffin embedded, and 5 micron-thick sections were processed for immunohistochemical staining for ribosomal S6 protein and its phosphorylated pS6 counterpart following the method as previously described [33].

### Statistical analysis

All results were expressed as the mean ± SEM. The differences in means of groups were determined by 2-tailed Student's *t* test. The minimum level of significance was set at p<0.05.

## Results

**Figure 1:**
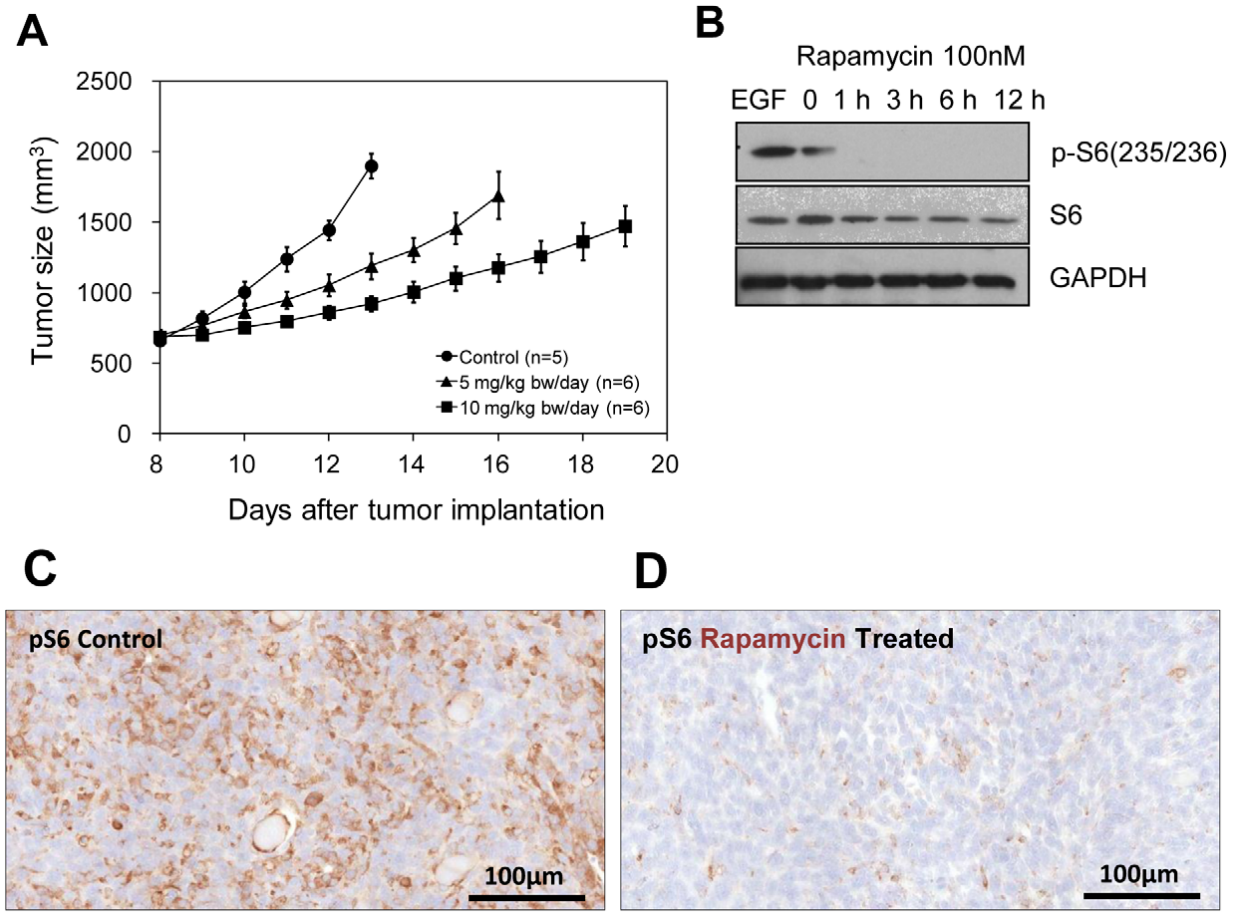
Effect of rapamycin on SCC tumor growth and mTOR signaling pathway. (A) Tumor sizes of the SCC tumors in the mice leg treated with vehicle (control, •), 5 mg/kg bw/day (▴), and 10 mg/kg bw/day (▪) rapamycin. (B) Western blot analysis of S6 protein expression and the abundance of its phosphorylated form in SCC tumor cells treated with rapamycin (100 nM). (C, D) Immunostaining of pS6 in SCC xenograft of control and rapamycin treated (10 mg/kg bw/day, 2 days).

To evaluate the effect of rapamycin treatment on SCCVII tumor growth, tumor sizes of a control group of tumor bearing mice and two groups of mice treated daily at 5 and 10 mg/kg bw/day (n = 5–6) were monitored. Rapamycin treatment was initiated 8 days post tumor cell inoculation in the right hind leg. A significant delay in tumor growth dependent on rapamycin doses was noticed in agreement with previous reports (Figure 1A) [11]. These results suggest that the SCCVII implants in C3H mice were sensitive to rapamycin as evidenced by the tumor growth inhibition.

Monitoring the accumulation of the phosphorylated form of the ribosomal S6 protein (pS6), which is the most downstream target of the mTOR pathway, can provide an exquisite surrogate marker to follow mTOR activity. In cultured SCCVII cells exposed to rapamycin (100 nM) for different times (0–12 h), an early decrease in p-S6 was noticed (1 h) while total S6 levels remained unchanged (Figure 1B). GAPDH was used as loading control. As SCCVII cells demonstrated sensitivity to rapamycin in vitro, corresponding xenografts were also assessed by immunohistochemistry for the status of pS6. As shown in Figure 1C and D, a significant decrease in immunoreactivity to the phosphorylated form of S6 was noted in the rapamycin-treated mice compared to untreated controls, demonstrating that rapamycin achieved its molecular effect in vivo. These results support the results shown in Figure 1A that the molecular target of rapamycin in SCCVII cells is being effected which is responsible for the tumor growth delay.

**Figure 2:**
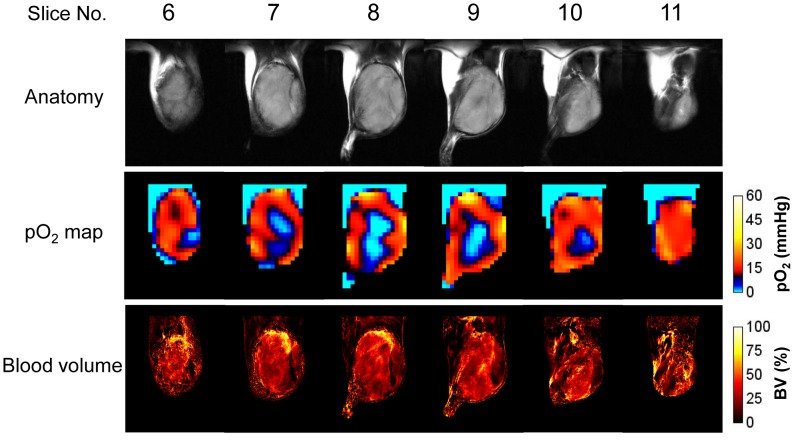
Anatomy, pO_2_, and blood volume images of SCC tumor. T_2_-weighted anatomical image (top) of a SCC tumor-bearing mouse, and the corresponding pO_2_ maps (middle) and blood volume images (bottom) measured by EPRI and MRI. The adjacent center six slices of the 3D images were displayed, and the every slice has 2 mm thickness.

Based on observations that rapamycin treatment in SCCVII tumor bearing mice elicits a tumor growth delay correlating with a decrease in the mTOR dependent signaling markers, we next conducted non-invasive imaging experiments to longitudinally monitor tumor oxygen status, tumor anatomy, and tumor blood volume in control and rapamycin treated mice with SCCVII implants by using EPRI and MRI. EPRI and MRI have been recently shown to have the capability to serially and non-invasively assess changes in tumor pO_2_ and microvessel density as a function of tumor growth or during a treatment course [19], [21], [23], [31]. Figure 2 shows results from such as an experiment with six adjacent slices of a vehicle-treated control tumor in leg on 12 days after tumor implantation, each 2 mm thick displayed for T_2_-weighted anatomy (top row), pO_2_ maps using the oxygen sensing EPR tracer Ox063 (middle row), and blood vessel density using the blood pool T_2_* contrast media USPIO (bottom row). The data presented show the capability of the imaging techniques to non-invasively obtain that pO_2_ distribution and microvessel density which show significant variation across the tumor.

**Figure 3:**
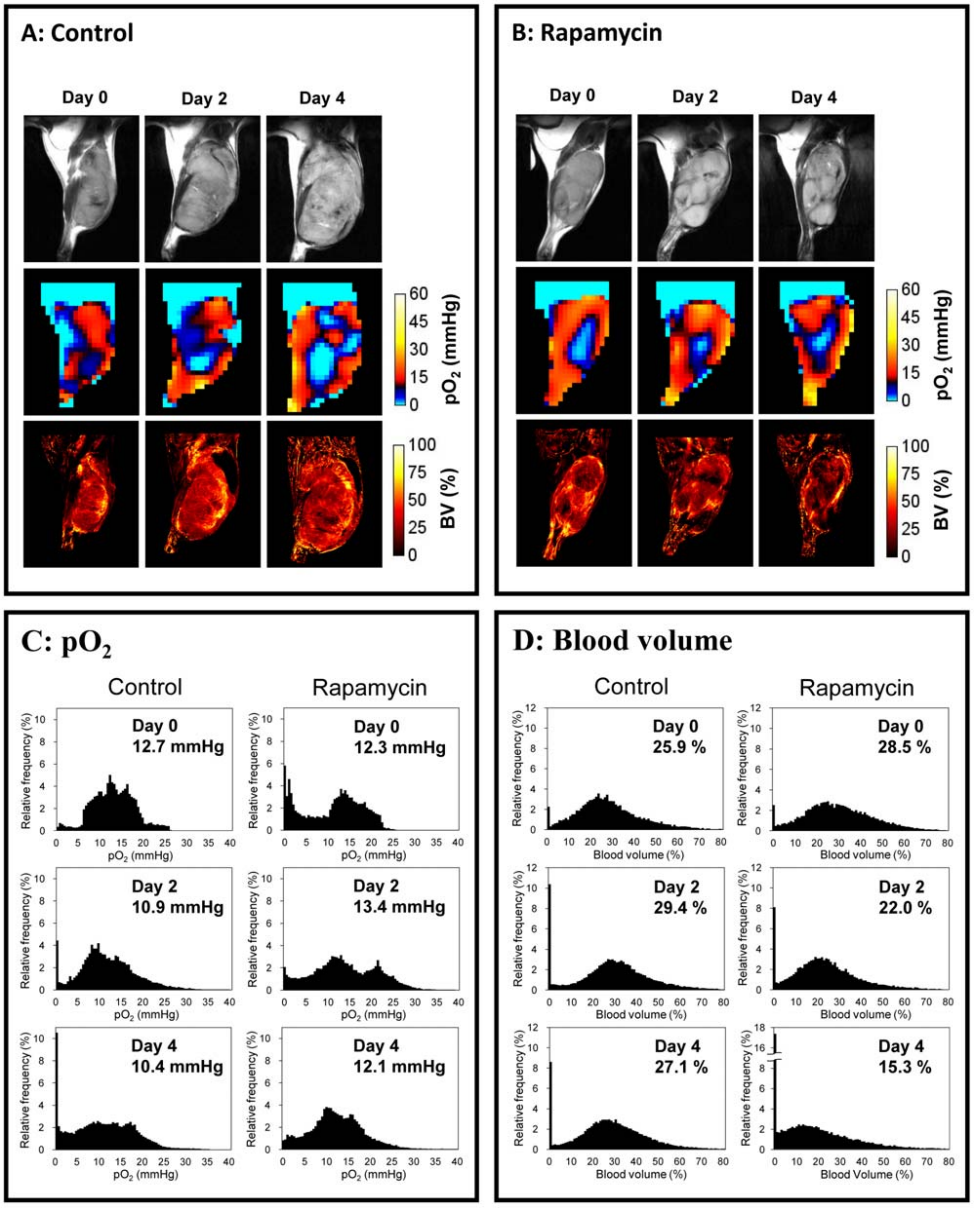
Effect of rapamycin treatments on tumor pO_2_ and blood volume. Anatomy, pO_2_, and blood volume images of SCC tumor in mice leg treated with vehicle (A) and 10 mg/kg bw/day rapamycin (B). The center slice of each 3D image is displayed. Treatment was initiated 8 days after tumor implantation (Day 0). The images of day 0 were obtained before beginning of the treatments. (C, D) Frequency histograms of pO_2_ and blood volume in the SCC tumors of (A) and (B). The values indicate median pO_2_ and blood volume in the tumor region.

Figure 3 shows results from longitudinal experiments from a representative control mouse and rapamycin treated mouse. Figure 3A shows the center slice of anatomy, pO_2_ and blood volume in the SCCVII tumor bearing mouse receiving vehicle as control on days 0, 2, and 4 (day 0 is 8 days after tumor implantation, on which the imaging study and the treatment was initiated). There were a lot of blood vessels observed throughout the tumor even on day 0, indicating angiogenesis already occurred on 8 days after tumor implantation. As expected, mice receiving no treatment exhibited increases in tumor size with the associated neovascularization supporting the tumor growth but with increasing hypoxia. Results from the mouse treated with rapamycin are shown in Figure 3B. It can be seen that a significant retardation of tumor growth (Figure 3B, top row) was accompanied by loss of tumor blood volume (Figure 3B, bottom row), and the extent of hypoxia did not increase in contrast to the control. While median values of pO_2_ provide a global assessment, histograms from the image data reveal additional information. Therefore, the results of figure 3A and B were analyzed by converting the pO_2_ images and blood volume images as frequency histograms (Figure 3C and D). The frequency histograms of tumor pO_2_ in the control mouse show a prominent shift leftwards on day 2 and day 4 compared to day 0 with a significant increase in the number of voxels having pO_2_ values below 5 mmHg (Figure 3C). On the other hand, in the rapamycin treated mouse, a significant reduction in frequencies below 5 mm Hg was observed with an appearance of a second peak around 22 mm Hg, indicating that a significant increase in the overall tumor oxygen status occurred on day 2 of rapamycin treatment. The peak around 22 mm Hg decreased on day 4 but the frequencies below 5 mm Hg noticed on day 0 did not return. A dramatic decrease in tumor blood volume in rapamycin treated mice was noticed on day 2 and 4 whereas it slightly increased in the control mouse (Figure 3D) Such behavior of transient increase in pO_2_ with decrease in microvessel density after treatment was commonly noticed in the case of antiangiogenic agents and this phenomenon attributed to neovascular normalization [15], [16], [19].

**Figure 4:**
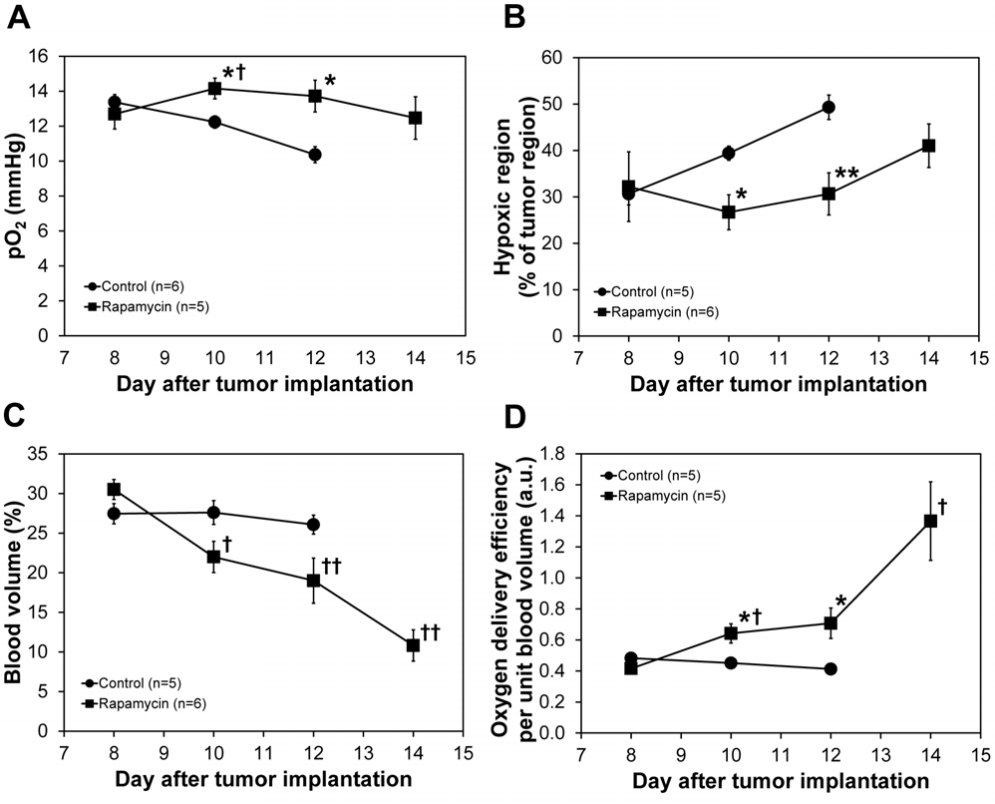
Changes in tumor oxygenation and blood volume. Median pO_2_ values (A), percentage of hypoxic fraction (B), and mean blood volume (C) in the control and rapamycin treated SCC tumors. The values are average of 5 or 6 mice and error bars represent standard deviations. Oxygen delivery per unit blood volume (D) was calculated by dividing tumor pO_2_ by blood volume. * p<0.05 as compared with control, ** p<0.01 as compared with control, † p<0.05 as compared with rapamycin day 8, †† p<0.005 as compared with rapamycin day 8.

The longitudinal changes in median tumor pO_2_ values in groups of control and rapamycin treated mice are graphically displayed in Figure 4A. It can be seen that while the median pO_2_ values were similar in the two groups on day 0, rapamycin treated mice show higher tumor pO_2_ values on days 2 and 4. The median pO_2_ in the rapamycin treated group showed a small decrease on day 6, but it was at the same level as that on day 0. Similarly, the hypoxic fraction (fraction of tumor with pO_2_<10 mm Hg) showed that rapamycin treated mice exhibit hypoxia to a lesser extent compared to control, untreated mice (Figure 4 B). Figure 4C shows the fractional blood volume in the tumors as a function of time. A significant decrease in blood volume in rapamycin treated mice compared to untreated mice was noticed on day 2. Continuing rapamycin treatment caused a further drop of blood volume on day 4 and day 6. An empirical analysis of tumor oxygenation status obtained from EPR imaging and the blood volume from MRI was done by obtaining the ratio of tumor pO_2_ with the fractional blood volume and plotted as a function of time and the results are shown in Figure 4D. The results show that oxygen delivery per fractional tumor blood volume in rapamycin treated mice was significantly more efficient than in control group of mice.

In order to investigate the underlying mechanism(s) associated with the observed improved tumor oxygenation, we carried out DCE-MRI study with Gd-DTPA as a contrast agent. It is well known that Gd-DTPA uptake is influenced by both tumor perfusion and vascular permeability. By considering only the initial rate of the Gd uptake the effects of changes in permeability on uptake can be minimized [39], [40]. Area under the curve (AUC) of Gd-DTPA concentration in the SCC tumor calculated from the DCE-MRI results of initial 1 min after injection was 40% larger in 2 days rapamycin treated group (0.188±0.017 µM, n = 5) compared to non-treated control group (0.134±0.025 µM, n = 5), indicating the improvement of blood flow in the SCC tumor by rapamycin treatments.

**Figure 5:**
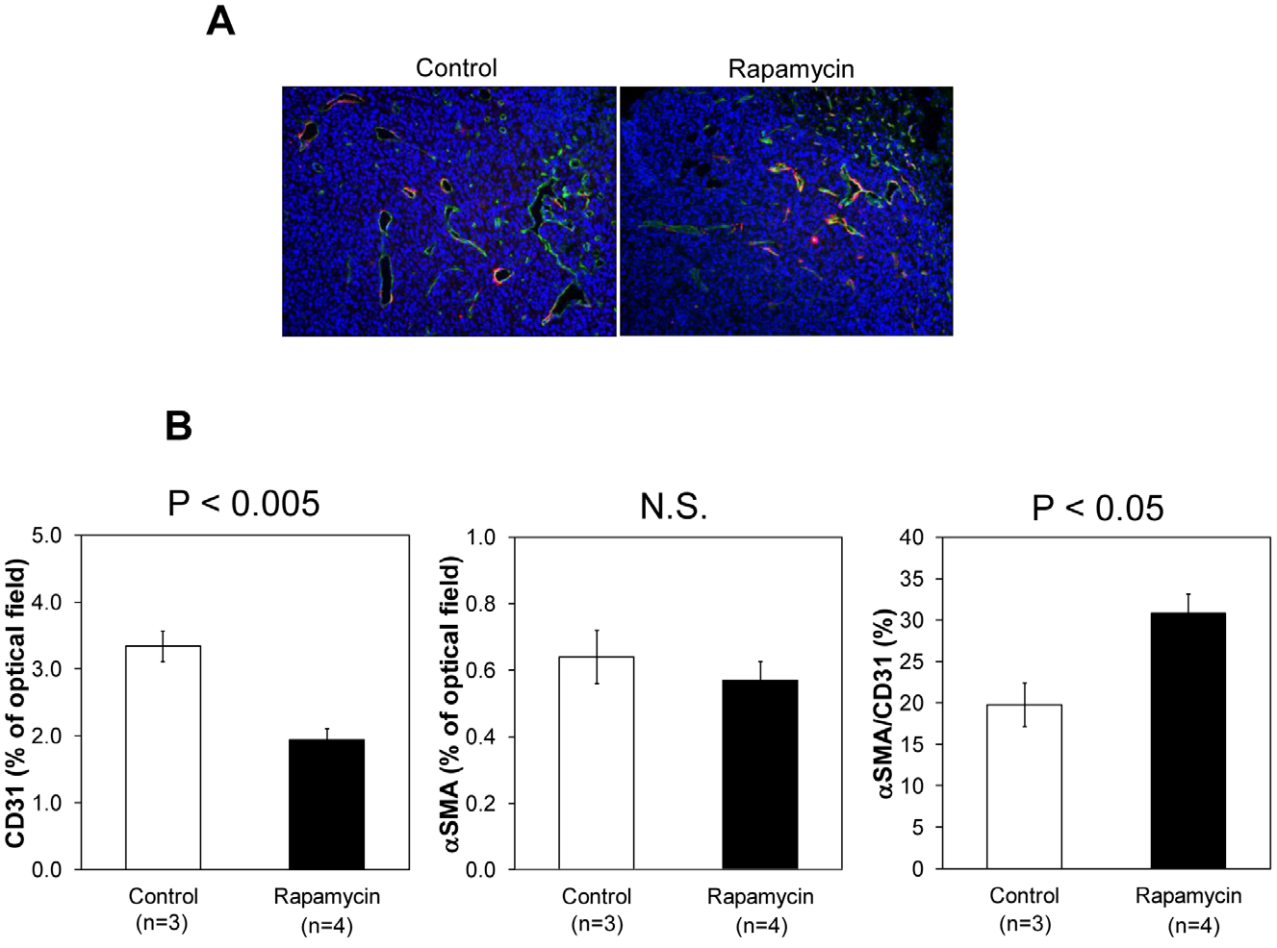
Immunohistochemical analysis of CD31 and αSMA in SCC xenograft. (A) Representative images of control and rapamycin treated SCC xenograft. Green is CD31, red is αSMA, and blue is DAPI. (B) Percentage of CD31 and αSMA in the SCC tumor of control (n = 3) and rapamycin treated (n = 4, 10 mg/kg bw/day, 2 days) mice.

Independent microscopic evaluation of tumor vasculature in control and rapamycin treated mice was carried out from tumor sections stained with CD31 (green) for microvasculature and αSMA for pericyte coverage (red) (Figure 5A). A significant decrease (* p<0.05) in tumor blood vessel density was noticed (Figure 5B) in rapamycin treated mice compared to untreated mice in agreement with the blood volume assessment from MRI experiments. When the histological data was quantitatively analyzed, it was found that the blood vessel density decreased by ∼30% 2 days after treatment with rapamycin. On the other hand, there was a small but not significant decrease in αSMA staining in tumors of rapamycin treated mice. The results shown in Figure 5 are consistent with the observations made by Lane et al [34] where the mTOR inhibitor RAD001 was more effective in reducing mature vessels with effective αSMA coverage than the anti-angiogenic agents tested.

**Figure 6:**
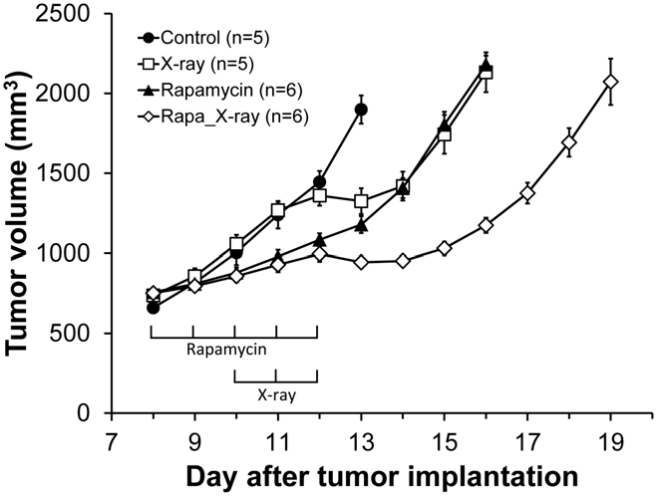
Effect of combination of rapamycin and X-irradiation on tumor growth. Growth kinetics of SCC tumors implanted in mice leg, treated with vehicle (control, •), X-irradiation (5 Gy/day, 3 days, □), rapamycin (10 mg/kg/day, 5 days, ▴), and rapamycin and X-irradiation (◊).

In order to examine if the pO_2_ increase by rapamycin treatment enhances outcome of radiotherapy, four different groups of tumor bearing mice (control; X-ray: rapamycin; rapamycin+X-ray) were monitored for tumor growth delay (Figure 6). Both mono-therapy of 5 days rapamycin treatment (closed triangles) and fractionated 5 Gy×3 days X-irradiation (open squares) suppressed tumor growth for 2 days. Combination of rapamycin and X-irradiation resulted in 5 days tumor growth delay (open diamonds). The “more than additive” growth delay may suggest the enhanced outcome of radiotherapy during vascular normalization window of rapamycin which transiently increases tumor pO_2_.

## Discussion

Increasing evidence supports a strong role for the mTOR complex as a critical regulator of cellular metabolism, growth, and proliferation [35], [36]. In carcinomas such as SCCVII, this pathway may be an early and widespread event independent of p53 status making this an important downstream target for therapy for mTOR inhibitors such as rapamycin and its analogs [7]–[10]. The mTOR pathway, being a part of the PI3K/Akt is considered as a key determinant in tumor angiogenesis through the expression of hypoxia related genes VEGF [13], [37]. Rapamycin and its analogs (rapalogs) target the mTOR pathway and induce cell death, autophagy and also exert antiangiogenic and antivascular effects in solid tumors [11], [14], [34], [37], [38]. Additionally, in preclinical models, rapamycin was shown to be an effective radiation sensitizer in vivo [20]. The results from the present imaging study provide non-invasive evidence for the rapamycin-induced loss in blood vessel density, but unexpectedly, we observed a concomitant increase in tumor pO_2_.

The antiangiogenic effects of rapamycin were first observed using a dorsal skin-fold chamber model using tumor implants in mice [37]. The antiangiogenic effects were attributed to decreased production of VEGF and resistance of endothelial cells to VEGF stimulation. Further studies used the rapalog RAD001 and compared its effects with known antiangiogenic agents [34]. The results showed that RAD001 was found to be associated with decreasing the tumor vessel density and the maturity of the tumor vessels, whereas the antiangiogenic drug vatalanib was found to impact only the microvascular density but not the vessel maturity consistent with this class of drugs which impact the VEGF/VEGFR complex [34]. On the other hand, Zhang et al. reported that the αSMA level, a marker of mature pericytes, increased in rapamycin treated tumor compared with non-treated tumor [14]. Other studies have shown that radiation induces activation of mTOR pathways in the tumor endothelial cells making them more sensitive to response with rapamycin [20]. However, a more recent study using a retro-inhibition approach found that HNSCC cells and not the tumor microenvironment as the target for rapamycin activity and that the anti-angiogenic effect is a likely downstream consequence of mTOR inhibition in cancer cells [13].

Imaging of properties intrinsic to tumor physiology such as tumor pO_2_ and tumor microvessel density made it possible to sequentially follow rapamycin induced changes during the course of treatment non-invasively and sequentially during the treatment course [19], [21], [23]. The key finding in the present study pertaining to the rapamycin effect on tumor physiology is that the tumor microvessel density, when monitored longitudinally showed a significant decrease whereas a transient increase in tumor pO_2_ was found followed by onset of hypoxia (pO_2_<10 mmHg). It should be noted that the USPIO-based blood volume assessment may overestimate the values in tumors because of their leakiness compared to normal tissues [31]. The observations from histological experiments were in agreement with the imaging observations. The rapamycin-induced decrease in CD31 staining was found to be in agreement with imaging experiments where a loss in microvessel density was found. However, there was a small but non-significant decrease in staining of αSMA which reflects the retention of the integrity of the pericyte coverage of the tumor vasculature after rapamycin administration. These results indicate that rapamycin treatment pruned immature blood vessels rather than mature blood vessels. It is expected that these changes in tumor microvasculature can cause improvement of blood flow, a phenomenon known as vascular normalization. The transient increase in the pO_2_ by rapamycin treatment can be attributed to the increased blood flow in the tumor, which was demonstrated by a 40% increase in tumor initial uptake of Gd-DTPA 2 days after rapamycin treatment in the DCE-MRI study.

The identification of transient improvements in tumor oxygenation 2 days after rapamycin treatment provides an opportunity for chemoradiation modalities where radiation therapy can be timed to take advantage of increases in tumor pO_2_ to elicit improved response [20]. The results in the present study show enhancement in tumor radioresponse by rapamycin treatment (Figure 6). This data suggests that the transiently increased level of median tumor pO_2_ in rapamycin treated mice compared to the day matched control group may be responsible for the observed effect of radioresponse with combination treatment. The relatively smaller effect of radiation with rapamycin (additive), in contrast with the observed synergistic effect of radiation with sunitinib in the same tumor xenograft [19], may be explained in terms of the relatively smaller magnitude difference in tumor pO_2_ in rapamycin treated group to the day matched control (∼2 mm Hg) compared to the greater difference in tumor pO_2_ in sunitinib treated group to the control (∼5.5 mm Hg). The significant synergy with mTOR inhibitors including rapamycin and radiation reported by Shinohara et al [20] may point out the characteristic influences of the microenvironment of each tumor type as pointed out in other studies where the synergy was attributed only to rapamycin targeting the enhanced activity of signaling pathways controlled by mTOR in the host endothelial cells [41]. Recent studies with a dual inhibitor of the PI3K and mTOR pathway found that the period of vascular remodeling is relatively more sustained than that observed with anti-angiogenic drugs resulting in substantial therapeutic gain [42]. These studies point to the importance of longitudinally monitoring such changes to realize maximal efficacy in combined chemo-radiation treatments. Imaging studies of the tumor microenvironment can establish a strategy in preclinical models to identify an optimal treatment schedule to realize enhanced response to combination treatments.

In summary, results from the current study show that molecular imaging techniques provide an opportunity to serially monitor changes in tumor physiology non-invasively and quantitatively and identify subtle physiological changes in response to rapamycin treatment. Therefore these techniques have the ability to provide valuable non-invasive biomarkers which predict treatment outcome and also identify temporal windows where radiation therapy can be advantageously combined to elicit improved response.
